# SAMQA: error classification and validation of high-throughput sequenced read data

**DOI:** 10.1186/1471-2164-12-419

**Published:** 2011-08-18

**Authors:** Thomas Robinson, Sarah Killcoyne, Ryan Bressler, John Boyle

**Affiliations:** 1 Institute for Systems Biology, 401 Terry Ave N, Seattle, WA 98109 USA

## Abstract

**Background:**

The advances in high-throughput sequencing technologies and growth in data sizes has highlighted the need for scalable tools to perform quality assurance testing. These tests are necessary to ensure that data is of a minimum necessary standard for use in downstream analysis. In this paper we present the SAMQA tool to rapidly and robustly identify errors in population-scale sequence data.

**Results:**

SAMQA has been used on samples from three separate sets of cancer genome data from The Cancer Genome Atlas (TCGA) project. Using technical standards provided by the SAM specification and biological standards defined by researchers, we have classified errors in these sequence data sets relative to individual reads within a sample. Due to an observed linearithmic speedup through the use of a high-performance computing (HPC) framework for the majority of tasks, poor quality data was identified prior to secondary analysis in significantly less time on the HPC framework than the same data run using alternative parallelization strategies on a single server.

**Conclusions:**

The SAMQA toolset validates a minimum set of data quality standards across whole-genome and exome sequences. It is tuned to run on a high-performance computational framework, enabling QA across hundreds gigabytes of samples regardless of coverage or sample type.

## Background

The growth in high-throughput sequencing means that there is a need for standardized, robust and scalable tools to perform quality assurance tests on the resulting data. The QA tools need to be extensible and are required to do more than check for instrument specific problems. The tools need to be able to check for both adherence to known standards and also to identify problems that may have arisen in sample preparation.

This paper introduces such a tool, built for analysis of data from The Cancer Genome Atlas (TCGA). This is the largest cancer genomics study to date, characterizing thousands of patients across 20 different cancers. The potential to discover new mechanisms and therapeutics from such a large-scale project is hugely important to the cancer community. However, the scale of the genomic data being generated by TCGA alone has already outpaced gains in computational power (associated with Moore's Law) making available analysis tools unusable on standard hardware. New sequencing technologies and the increasing interest in population-scale genomic analysis will only exacerbate the computational problems. Within low-throughput genomic data many errors can be characterized simply using the Sequence Alignment/Map (SAM) specification [1] and tools are available for reading and manipulation of sequence files. However, as the size and scale of genomic data increases these tools often struggle to perform at the necessary speeds (or at all). SAMQA provides quality checking using a high-performance computing framework to quickly and automatically capture and report errors across population-scale data. SAMQA has been designed to use the latest advances in high performance computing to ensure it is able to scale from gigabytes to petabytes of genome sequences regardless of sequencing platform, depth of coverage or data type.

An important step in the analysis of these large-scale genomic data is verification of a minimum standard of quality. With large scale sequencing projects there are a number of data quality issues that must be addressed, as biases are introduced at multiple levels. In population-level investigations data can significantly influence secondary analysis [2], especially when looking at cancer data where genomic variation is high and largely uncharacterized. Within TCGA alone, issues affecting the quality of sequence data range from sample collection to selection of sequence alignment tools:

*• Biological sample collection*. Despite the use of standard clinical methods and procedures, the samples may lack consistent purity [3] resulting in highly variable data within the population.

*• Laboratory methods*. Coverage discrepancies can result from the use of specific genome amplification methods [4] (e.g. GenomiPhi, Repli-G, PCR), potentially differentially representing areas of the genome.

*• Instrumentation bias*. Sequencing instruments are known to introduce specific anomalies, from Illumina's G-C bias [5] to the 454 Life Sciences [6] errors in regions that vary too much from the reference sequence.

*• Bioinformatics*. Multiple tools and tool versions are available to align reads to a reference genome. Within TCGA versions of BWA [7], Bowtie [8], GATK [9], Maq [10], and BFAST [11] are all used for sequence alignment. This results in data with a variety of computational errors as reads are matched and scored.

The next section discusses the types of tests that SAMQA uses to identify technical errors in the structure of the sequenced read data and biological errors which are assessed by extracting features which correspond to likely anomalies from the data. The Results section introduces how the high-performance computing system has been designed to scale to the required level. The Results section gives an example QA analysis that was undertaken across approximately 400 genome files.

## Implementation

When attempting to classify anomalous data sets, we must begin with the question, "What constitutes an anomaly?" This is a difficult question to answer when we expect our data sets to be highly divergent as: the samples can be gathered from different cohorts and populations; they typically will contain a high number of polymorphisms and structural variations which differ from the references sequence(s); and they often contain batch effects due to sample collection and instrumentation differences.

Our approach has been to classify anomalies in sequence data sets relative to individual reads within a sample. The detection of these can be completely automated through the use of technical specifications, and allow for the inclusion of a level of data and biologically specific tests which require expert input.

### Technical Tests

The default technical tests are defined using structural components of the Sequence Alignment/Map (SAM) specification dated 2010-12-11 (version v1.3-r882). These tests generally include verifications of the SAM file format itself (see Table 1). These errors are clearly defined through the use of standard metrics such as: it contains all reads that map beyond the length of a chromosome; contains an invalid CIGAR value; or contains zero-length reads. As these tests are defined through a well-known standard (SAM), and implemented in the Picard toolset from SAMtools [12,13], they are also fully automatable.

**Table 1:** SAMQA Technical Tests

Technical Tests	Examples
Header Consistency	Consistency of chromosome identifiers, presence of read groups and sequence dictionary, valid reference sequence.

Specification Adherence	Correct regular expression for flag value, query name, mapping position, mate mapping position, inferred insert size, etc.

Functional Relationships	Incorrect flags set (e.g. Reads not mapped in a proper pair, with proper pair flag set), adjacent indel present in CIGAR value, invalid or missing tag NM, and functional dependent fields do not align or are invalid.

This list includes errors that are defined by the SAM format. Errors may be introduced at multiple levels as described in the "Examples" column. These tests are run in SAMQA using the Picard toolset from SAMtools.

### Biological Tests

The biological plausibility of the test results is typically judged through expert analysis. In validation all features of the data that are highly unlikely in a biological context (e.g. valid reads, structural variation) are identified. The formal determination of implausibility requires a confidence threshold to offer meaningful results. This threshold determines for each feature whether a read should be considered highly erroneous (see Table 2) requiring some level of involvement from a domain expert (e.g. errors in clinical cancer data vs. errors within yeast population sets). When the tool is run automatically the threshold can be used to assign pass/fail flags to each of the sequence files.

**Table 2:** SAMQA Biological Tests

Biological Tests	Inclusion Criteria
Mapping quality	Low Phred-adjusted mapping quality score

Read length	Shortened read lengths for a given sequencing technology

Read count	Low aggregate number of reads for a given sequencing technology

Read frequency	Low number of reads for a given set of kilobase regions

Coverage	Low coverage for a given read group, chromosome, or kilobase region

Structural variations	High numbers of localized structural variation

Anomalous sequence data	Instances of "random" chromosomes from human assembly [8]

Population estimates of structural variation	Very high projected structural variation across different platform units

Read group correlation	Low mapping quality correlation for megabase regions, across read groups Low coverage correlation of megabase regions, across read groups

These tests extract useful, biological features from the data for expert analysis. Other extraction tools (e.g. detection of polyadenylation within individual sequences, determinants of the feature-dimensional "shape" of the data, as through multidimensional Bayesian analysis) may be added as appropriate to the data or downstream analysis requires.

At its core, SAMQA is a series of tools that determine if a sample has syntactic errors and uses a series of heuristic measures to identify likely biologically improbable anomalies. The system is designed to be extensible so that further tests can be easily added. In implementation, SAMQA is two sets of tools that process Binary Alignment/Map files using a defined standard (SAM) and expert analysis.

### High Performance Computing

Even for low coverage sequence files, traditional approaches to processing these data on a local machine are highly limited relative to the enormity of input data from sequencing methods. The SAMQA system has been designed to scale to meet the needs of investigations that may generate thousands of genomes. When high-performance computing (HPC) is required, the BAM processing layer is handled by the Hadoop-BAM project [14], with minor modification to allow BAM sequence indexing to occur entirely within the Hadoop Distributed File System (HDFS), a storage solution that spans disks in a cluster to provide one large, distributed file system [15,16].

The MapReduce framework [17] was selected due to the highly partitionable nature of the input data sets, and the relative ease of adapting our workflow to the MapReduce paradigm when searching for read-based structural errors. Furthermore, as all operations that we define are commutative and associative, we can make use of Hadoop's intermediary *Combine *operation when transitioning between the *Map *phase and *Partition*, *Shuffle*, and *Sort *steps of Hadoop's implementation of the framework. By specifying an intermediary *Combiner*, we can reduce network and processing overhead by performing an immediate *Reduce *over the output data sets of mappers on each cluster machine. For example, using a small, 80-core cluster running OutputCoverage (our tool to generate coverage totals per kilobase region), the use of a combiner means the difference between three minutes and nearly 90 minutes of analysis over the same BAM file (see Table 3), utilizing the same job across the cluster. This significant difference is due to the sheer volume of data reduction achieved by collapsing a large number of map outputs into a very small number of outputs from each *Combiner*.

**Table 3:** Run Time for SAMQA on Hadoop

Validation Test	One Patient, aggregate time in minutes (over 23GB)	Ten Patients, aggregate time in minutes (over 405GB)	One Hundred Patients, aggregate time in minutes (over 3,904GB)
Mapping Ratio Test	1.10	6.98	66.91

Sequence Validation	5.40	66.11	621.54

Read Group Validation	8.40	86.38	670.79

Mapping Quality Test	9.03	57.27	706.02

Read Statistics	11.28	67.49	753.00

File Structure Validation	15.10	59.57	700.67

Chromosome Check	63.15	102.02	768.37

Pearson Coverage Correlation	279.41	280.50	3211.33

Pearson Mapping Quality Correlation	702.28	484.92	3327.94

This table lists required time to completion at multiples of ten samples over an 80-core computational cluster running Hadoop. Each validation step includes a full *Map-and-Reduce *pass. All times are given in aggregate time to completion, assuming each file is scheduled for analysis after the previous one completes. The files selected are listed in additional file 2. File size is the only consideration for speed comparisons.

Our selection of the MapReduce framework is notable because, even for jobs bound by linear or linearithmic time and space, data sets and analysis can quickly prove unwieldy at the tera- and petabyte scale (see Table 4). MapReduce's strengths in data processing, when coupled with Hadoop's strengths in data and computational locality, task delegation, and aggregation strategies over key-indexed information, provide significant constant time improvements, with a near-linear speedup bounded only by the number of nodes in a compute cluster. In the context of a *Mapper*-only job, we see improvements in constant processing time plus linear data marshalling time associated with Hadoop distributing tasks to individual machines. In the case of a job that also defines a *Reduce *operation, improvements are linearithmic with its primary overhead attributable to distributing tasks and the *Partition, Shuffle*, and *Sort *operations performed by Hadoop.

**Table 4:** Run Time SAMQA on Hadoop vs. Standard Server

SAMQA Run	Single Core Server (in hours)	Hadoop Cluster (aggregate in hours)
**1 Sample (23GB)**	1460.22	18.25

**10 Samples (405GB)**	1615.00	20.19

**100 Samples (3904GB)**	14435.42	180.44

The SAMQA toolset consists of eleven different technical and biologically relevant tests run over each BAM file. Using the Hadoop cluster and data as referenced in Table 3, all of the tests can be run in parallel decreasing the overall time for analysis (files used for this test are listed in additional file 2).

**Figure 1:**
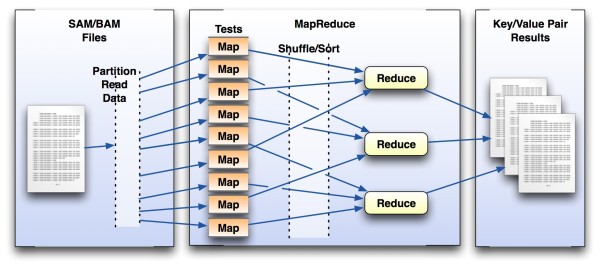
**MapReduce Framework**. MapReduce provides a generic framework that enables rapid, parallel analysis of partitionable data. Each *Mapper *runs a single test (e.g. OutputCoverage) across a section of the data from a single sequence file. The *Reduce *phase (if specified) can do additional analysis across aggregated data or *mapper *results and output information that can be used by external tools (as in Figure 2).

All feature extraction tools are implemented as separate MapReduce jobs, which operate on the complete data sets as described (in Figure 1). While this results in referencing the same read data multiple times (once for each job), the speed and performance of HDFS trivialize this operation through data and cache locality. As a result of these systems working in tandem, the toolkit is extremely robust, fast, easy to understand, and easy to extend. We believe that these factors are vital to the extensibility of SAMQA as new analysis types are requested and added to the tool.

Each feature extraction tool outputs a series of key-value pairs in a human-readable and machine-parsable text format. Each key-value pair describes some feature of the data as defined by the tool being run. For example the pair < BIN_example_0_COVERAGE, 9.4126293> has the compound key BIN_example_0_COVERAGE describes the coverage for the kilobase region 0 for our example sequence, and the value 9.4126293 denotes coverage over this region. While somewhat terse in their contrived meaning, all compound keys output by SAMQA are fully representative and uniquely identifiable. This simple representation is designed to make it easier to integrate with downstream analysis tools while remaining extensible to the operating standards of those wishing to integrate the tool into their own workflow.

For all *Mapper*-only jobs, the mappers are used to perform parallel computations against isolated, atomic data fragments. In a MapReduce operation, each *Mapper *performs input pre-processing, the *Combiner *(if specified) aggregates these intermediate results, and the *Reducer *performs each final calculation. This is especially vital for any tests across the entire input data such as Pearson correlations or calculations of coverage.

**Figure 2:**
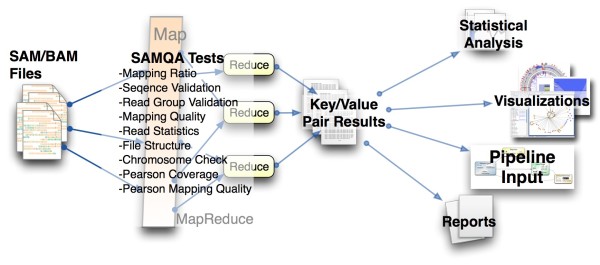
**SAMQA System**. The SAMQA system can run either on a single machine, a distributed cluster, or across heterogeneous servers using HDFS. The tests are run against the stored BAM/SAM files and the output is generated as key/value pairs. The results can be explored using the generated reports and visualizations, or through R. SAMQA can also operate in batch mode so that it can be run as part of an automated pipeline.

While SAMQA provides a clean pass or failure for invalid reads, it provides no additional facilities to process these key value pairs (see Figure 2). We leave this job to post-processing and data mining tools better equipped to the task, which may operate on a vastly reduced output data set relative to the size of the original sample files. We have currently defined tools built on top of SAMQA that parse these key-value pairs in Python and additional visualization tools written in R. More details regarding setup and use of SAMQA as well as information about the output format can be found at http://informatics.systemsbiology.net/project/samqa

## Results

The SAMQA toolkit was developed to support work being undertaken at the Center for Systems Analysis of the Cancer Regulome, which is one of the TCGA Genome Data Analysis Centers.

**Figure 3:**
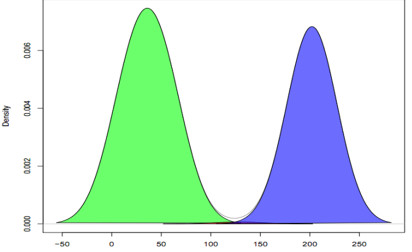
**Average Mapping Quality of COAD/READ Data**. Density plots of the average mapping quality for the individual COAD/READ files. The plot shows that the distribution of average mapping quality for the 236 file data set (listed in additional file 1) is bimodal based upon the mapping quality of two different sequencing platforms over exon data (Illumina and SOLiD).

**Figure 4:**
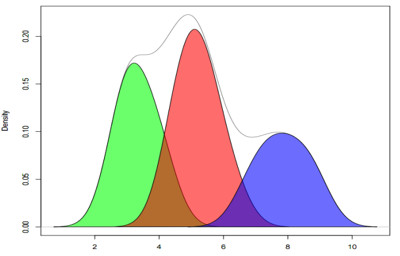
**Average Coverage of COAD/READ Data**. Density plot of the average coverage for the 236 COAD/READ files. The coverage of the files varies from 2× to 8×. The tool allows for the easy identification of files with similar average coverage. The separation shown here, while less clear than in Figure 2a, is also due to use of different sequencing platforms (Illumina and SOLID) over exon data.

The tool is run across all samples prior to secondary analysis. In a recent QA run on COAD/READ (Colon/Rectal Adenocarcinoma) samples the tool was used to analyze 324 exome and 42 full genome samples. The results of the technical tests are summarized in Table 5, and the results of the biological tests are shown in Figures 3 and 4 (SAMQA output shown in additional file 1). The tool automatically rejected those samples that failed the technical tests (e.g. six samples that contained only unmapped reads).

**Table 5:** COAD/READ SAMQA Results

Sample Group	Anomaly	Files affected
	"CIGAR should have zero elements for unmapped read"	150 files
236 COAD/READ exon capture sequence files.	Files were completely unpaired in sequencing	5 files
	Files contained unpaired reads	1 file

	"CIGAR should have zero elements for unmapped read"	48 files
48 COAD/READ whole genome files	"MAPQ should be 0 for unmapped read"	48 files
	"RG ID on SAMRecord not found in header"	18 files

The technical tests were run across 236 exon capture sequences files and 48 full genome sequence files for COAD/READ cancer samples from the TCGA project. The results identified problems with the files, and also identified 6 files that could not be used for further analysis. The mapping issues found in the whole genome and exon datasets are due to a documented issue within the alignment tools where BWA maps beyond the reference. The tool flags it as an error, but it is non-fatal to SAMQA. The files and SAMQA output are provided in additional file 1.

The biological validation tests are used to further explore the data. The exploration allows for the identification of files that have similar properties, which will result in batch effects (e.g. lower average quality scores or coverage - see Figures 3 and 4), as well as individual outliers. The tests themselves are output as a single file, and can be read directly into an analysis program. The supplementary materials contains the output for the default tests that have been run across both the COAD/READ samples, as well as Glioblastoma (GBM) and Ovarian (OV) cancer samples.

## Conclusions

SAMQA is a QA analysis toolkit that runs a series of tests over sequenced read data and is optimized for large numbers of files. The tests are for both verification of errors according to the SAM specification, and for assigning scores relating to the biological implausibility of structurally valid, dubious reads that relate to putative erroneous samples. It provides a simple, extensible, and robust framework built on top of Google MapReduce and Apache Hadoop, and is capable of processing large volumes of data quickly in a highly parallel manner. We have used the tool for analysis over data sets of OV, GBM, and COAD/READ cancer data from TCGA. The software can be used, with minimal extension, to provide useful analysis of any form of sequenced read data in SAM-defined formats.

We believe that this tool is valuable to the medical research and bioinformatics communities specifically, as it provides a sanity check and second opinion that release data sets are valid. The tool can be used as part of an automated pipeline, and the HPC system means that the tool can be run repeatedly on increasingly large files as investigations evolve. SAMQA also provides an improvement in standards for release quality, which we feel is valuable in a community that relies heavily on custom and highly vendor-specific technologies for sequencing and data processing. SAMQA is released as a free and open-source tool to the community.

## Availability and requirements

Project Name: SAMQA

Project home page: http://informatics.systemsbiology.net/project/samqa

Operating system: Platform independent.

Programming language: Java

Other requirements: Java 1.6 or higher, computational cluster or single server running Hadoop 0.20.2. Basic familiarity with Google MapReduce and Apache Hadoop. Further information on setting up and running a Hadoop cluster can be found in Hadoop's Quick Start guide [18], Cluster Setup guide [19] or in Tom White's book, *Hadoop: The Definitive Guide* [15].

License: Apache version 2.0

## Authors' contributions

JB conceived of the tool and participated in the design, testing and validation. TR designed and developed the toolset. SK participated in design, testing and validation and contributed equally with TR to the manuscript. RB participated in result validation. All authors approve of this manuscript.

## Supplementary Material

Additional file 1 **SAMQA COAD Output**. This provides the output of the SAMQA tool after a run over the 236 COAD data files. Click here for file

Additional file 2 **SAMQA Output for Hadoop Comparison**. This lists the files used for the test time comparison across Hadoop and the single-core server. Click here for file
